# Remote audits: a CRO virtual reality – what lessons have been learned in a time of unexpected isolation

**DOI:** 10.4155/bio-2021-0126

**Published:** 2021-07-22

**Authors:** Angela D Taylor, Marc M Twitty, Rafiq M Islam

**Affiliations:** ^1^Smithers Pharmaceutical Development Services, 11 Firstfield Road, Gaithersburg, MD 20878, USA

**Keywords:** audit, CRO, inspection, pandemic, quality assurance, remote, virtual

Have you ever walked into an office, and it was filled with papers all over the desk and drawers and just thought to yourself ‘this is a hot mess!’; then you proceeded to clean up the situation? First by organizing the paperwork into piles and then categorizing/sorting the piles to try to bring some sanity to the situation before even being able to file the paperwork away? Well, this is how the earliest days of remote auditing felt to many of us.

The days when an auditor would travel to a site to conduct an audit in-person and have documents presented in a well practiced, well-organized fashion are a distant memory. In retrospect, it seemed so much easier then and that was just a short while ago although the pandemic has made it seem much longer than that. While the purpose and events that take place during an audit have not changed, the process has become a bit more complex. The challenge now is to manage the audit virtually, across great distances, various time zones and countries through virtual meeting rooms, utilizing electronic, sometimes fickle means; while still completing the audit within the allotted timeframe with as little stress to both parties as possible may appear to be a daunting and difficult task. However, with a little preparation and the right tools, completing a successful remote audit is achievable under almost any condition.

None of us could have possibly known that the COVID-19 pandemic would last so long and be so disruptive to our work lives and that the new normal would no longer be to work onsite in an office space or to travel to a client, vendor or subcontractor to audit. Instead, we have had to adjust to working from home or deal with individuals who work from home, making activities that were typically easy, more cumbersome to execute. Some of the challenges faced with remote auditing are presented in the list below:

- Hardcopy documents not available in an electronic version;
- A lack of knowledge on how to operate the software tools (e.g., Zoom, Teams, etc.) used for the meeting and sharing of information;
- Unable to securely share proprietary documents and large files;
- There is no structure to the audit leading to chaos or so much structure that the audit is rigid and lacks flexibility;
- Video conferencing equipment malfunctions without an alternate plan;
- The agenda does not include ‘bio-breaks’, time for the participants to have lunch and such;
- The agenda does not dedicate time for auditor review where the auditor can review uploaded documents and the auditee can gather and complete requests;
- Time zone differences (e.g., USA: East vs West Coast; North America vs Europe or Asia) – agreement of a convenient time that works for all individuals involved in the audit;
- Key personnel are working remotely and rely heavily on onsite personnel for audit preparation;
- Conducting a virtual tour of the lab and facilities.

When hosting a remote audit, it is imperative to be respectful, flexible, patient and use good virtual etiquette. What is meant by virtual etiquette? Show video (yourself on camera) whenever possible during meetings. Mute yourself when appropriate, such as when you are typing or there is other noise in the background. Turn off the camera when doing any activity that would be viewed as distracting during the meeting. Do not talk over anyone, especially the host, it can be viewed as being aggressive and rude.

Like the hot mess mentioned above, be systematic and organized when hosting an audit. Know what your expectations are for pre-, during- and post-audit activities. The concept is the same as organizing (pre), categorizing (during) and filing (post). If there is a system in place, you are one step closer to having a successful audit.

## Pre-audit activities

When preparing for your virtual audit, have a plan of execution. This involves having a means to conduct the audit, having tools available to manage documents, understanding the logistics of how the audit should be executed and preparing a schedule to use as a guide for conducting the audit.

### Have a platform to use to conduct the audit

There are several platforms available for use to hold a virtual meeting. Some are easier to manage than others, each has its pros and cons. The auditee should decide which platform to use since the host will need to know how to navigate the meeting room. Prior to the audit, make sure that the host has the appropriate account access to use special features of the meeting room application. For instance, in one app, the use of session breakout rooms is only available for users with administrative access. Knowing what your requirements are for the meeting and discussing them before-hand with your IT department to ensure the proper level of access will save a lot of headaches downstream.

### Have your tools ready

Knowing what platform the audit will take place in is only the first step of preparation. The auditee must also have tools available for document sharing and presentation. The preparation of a remote audit is similar to that of an in-house audit; however, one must have a virtual space in which to share documentation. Make sure there is a portal or document share available where the auditee can securely load files to share with the auditor. We have utilized our company’s portal as well as the portal of the auditor. It has been more beneficial to use one’s company portal so access and capabilities within the portal can be controlled, allowing the auditee to manage the documents being shared.

Another tool that has become invaluable is a projector camera. Most participants are pleasantly surprised at the discovery of a live camera feed being used to show active in-house hardcopy documents such as training forms and equipment maintenance records. As stated earlier, you want to share an image of yourself with everyone on the call. To do this, make sure there is a camera connected to your computer before the audit begins. Next, have an nondisclosure agreement/confidential disclosure agreement in place before sharing documents with the auditor. The last tool to mention is one that is not readily thought of a company phone, tablet or similar video streaming device. This tool is needed to accomplish virtual tours of key spaces where the work is being performed.

### Share the logistics of how the audit will be executed

Decide how you, as the auditee, want the audit to be executed. Do you want to schedule multiple meetings for the meeting room, or do you want to have only one meeting throughout the entirety of the audit in which participants can enter and exit? As an auditee, we utilize the latter, create a single meeting room that is used by the participants for individual meetings. We have also found it important to populate a spreadsheet for requests prepared in advance to document the requests and track completion. Schedule a conference room as the base of operations for the duration of the audit. The conference room should be equipped with the devices needed to host the audit. Make sure the reservation includes time before and after the audit for preparation and closeout.

### Have a schedule

Make sure to have meetings/interviews with key individuals scheduled at least 1 week in advance. The auditor should have communicated their schedule for the audit at minimum 2 weeks in advance of the audit date. Create an internal audit schedule from the one submitted, segment each topic into a time slot and assign subject matter experts to each segment. Once there is agreement with the auditor on the audit schedule, calendar invitations should be sent to the subject matter experts to reserve their time.

## Working through the audit activities

When speaking about working through the audit activities, it means gathering documents prior to the audit, testing the systems and tools available by completing a walk-through and reaching out to participants and providing details or information that may be useful toward their segments. From the opening meeting to the closing meeting, the audit should be manageable.

### Gather audit documents

For any audit, onsite or virtual, it is key to have gathered the necessary documents as requested by the auditor. This list is provided with the schedule. Take this activity one step further and anticipate what the auditors may ask for and have the documents available. For an onsite audit, there is the freedom to leave the auditor in the conference room while he/she is reviewing documents to pull new requests. Once the item is found, it is quickly reviewed and then handed to the auditor to confirm receipt and review. For a virtual audit, the process takes longer as the item may go through several steps prior to reaching the auditor’s virtual hands. The document will still be retrieved and reviewed; however, it will also need to be scanned, saved to an audit folder and uploaded to the portal. Alternatively, if using the projector, the audit will be paused while the document is presented to the auditor, ensuring that the auditor is able to capture sufficient information. Having potential request documentation available, normally training files selected from personnel who worked on the project, equipment and systems validation records and facility maintenance will save time to pursue other activities. Documents requested from the auditors, including all standard operating procedures requested, should be provided at least 3 days prior to the audit so they can be reviewed before the start of the audit.

### Systems walk-through

Two system checks should occur before the start of the audit to test the tools. The first is an internal check to make sure the tools are hooked up properly and the operator knows how to use them. If a tour of the facilities is planned, the auditee should complete a dry run with the scheduled participants so everyone is on the same page for how the tour will be conducted. During the tour, do not move the camera around quickly as this can make the viewer dizzy and disoriented. When videoing, pan the room slowly and home in on key identifiers such as equipment IDs and calibration stickers.

The second system check is a meeting between the auditor and auditee to test connections, discuss logistics and work out the final details of how the audit will be conducted. Once both system checks have been completed, the auditee should feel more confident about the pending audit.

### Participant instruction

Reach out to the participants of each section regarding any observations made while reviewing requested documents so they may be prepared for potential questions that the auditor(s) may have. Also ensure that the participants are aware of the schedule, understand their role and are at ease with the logistics of the audit.

### Audit execution

The day of the audit, make sure to begin setup at least half an hour prior to the opening meeting start. Three methods of presenting/managing the audit have been used during our audits. First, confirm that the conference room computer system is operating as needed to host the virtual meeting. Log into the meeting as host and test that the projector is operating properly. Second, is having a work laptop available. This allows the host to have their day-to-day items open and to easily navigate between electronic audit materials for upload to the portal and/or present to the auditor. Third, is the tablet or phone that is used for facility tours. When operating multiple devices through the virtual meeting at the same time, be sure to mute all but one of the devices to avoid distractions, background conversations, noise and feedback.

Toward the end of every day, allow the auditors time to review their notes before meeting with them to recap the activities and requests from the day. Akin to onsite audits, once the audit is dismissed for the day, make every effort to retrieve the documentation that was requested and either have it ready to present or uploaded in the document share portal prior to the start of the meeting the next day. Making sure to stay on top of documenting and fulfilling requests is also important for the successful completion of the audit.

## Post-audit activities

Activities following a virtual audit will mirror activities following an onsite audit. The auditor will send an audit report with observations ranked by criticality and will provide a due date for responses. How the remote audit closeout differs from onsite audit closeout is in the management of the document portal. Prior to the end of the audit, discuss with the auditors and management if additional time will be needed for the auditors to view the documents in the portal before removing their access. This can be a standard amount of time or can vary according to how many of the requests are pending at the end of the audit. Remote audits move fast, there are more than likely going to be pending requests. Following the closing meeting, complete the open requests within the time period the portal will remain open.

## Summary

When first learning about remote auditing, an impression was formed that this would be a labor intensive, cumbersome process where requested documents would be provided to the auditor for review on the auditor’s schedule at the auditor’s worksite. With the use of online meeting rooms and other tools, and all parties becoming more fluent in navigating virtual meeting apps, remote auditing has reached a new level and has now become feasible even for auditing on a global scale. Remote audits have shown their utility and successfully achieve the objectives of the inspection on par with audits conducted in-person at the facility. The COVID-19 pandemic has required us to review our routine business practices and adapt to the challenges imposed by travel restrictions and work from home policies. Our industry is resilient and has responded accordingly. As a result, remote audits have become the new standard and will remain a viable alternative for conducting quality and compliance assessments into the foreseeable future. With the right tools, an open mind and a lot of flexibility; remote audits can be both a successful and an enjoyable experience.

